# MiR-92d-3p suppresses the progression of diabetic nephropathy renal fibrosis by inhibiting the C3/HMGB1/TGF-β1 pathway

**DOI:** 10.1042/BSR20203131

**Published:** 2021-09-03

**Authors:** Yuhua Zhang

**Affiliations:** College of Medicine, Jiangxi University of Technology, Nanchang 330098, Jiangxi, China

**Keywords:** C3, diabetic nephropathy, HMGB1, miR-92d-3p, renal fibrosis, TGF-β1

## Abstract

The pathogenesis of diabetic nephropathy (DN) has not been fully elucidated. MicroRNAs (miRNAs) play an important role in the onset and development of DN renal fibrosis. Thus, the present study aimed to investigate the effect of miR-92d-3p on the progression of DN renal fibrosis. We used qRT-PCR to detect the expression levels of miR-92d-3p in the kidneys of patients with DN. Then, after transfecting lentiviruses containing miR-92d-3p into the kidneys of a DN mouse model and HK-2 cell line, we used qRT-PCR to detect the expression levels of miR-92d-3p, C3, HMGB1, TGF-β1, α-SMA, E-cadherin, and Col I. The expression levels of interleukin (IL) 1β (IL-1β), IL-6, and tumor necrosis factor-α (TNF-α) in the HK-2 cells were detected through enzyme-linked immunosorbent assay (ELISA), and Western blotting and immunofluorescence were used in detecting the expression levels of fibronectin, α-SMA, E-cadherin, and vimentin. Results showed that the expression levels of miR-92d-3p in the kidney tissues of patients with DN and DN animal model mice decreased, and C3 stimulated HK-2 cells to produce inflammatory cytokines. The C3/HMGB1/TGF-β1 pathway was activated, and epithelial-to-interstitial transition (EMT) was induced in the HK-2 cells after human recombinant C3 and TGF-β1 protein were added. miR-92d-3p inhibited inflammatory factor production by C3 in the HK-2 cells and the activation of the C3/HMGB1/TGF-β1 pathway and EMT by C3 and TGF-β1. miR-92d-3p suppressed the progression of DN renal fibrosis by inhibiting the activation of the C3/HMGB1/TGF-β1 pathway and EMT.

## Introduction

Chronic kidney disease (CKD) is a heterogeneous group of disorders. According to the current U.S. Centers for Disease Control and Prevention (CDC) statistics, 37 million people in the United States are estimated to have CKD. In addition, CKD is more common in people aged 65 years or older (38%) than in people aged 45–64 years (13%) or 18–44 years (7%) [1]. The prevalence of type 2 diabetes (T2DM) shows an increasing trend yearly. Statistics from the International Diabetes Federation showed that the number of patients with T2DM may exceed 600 million by 2035. Diabetic nephropathy (DN) is one of the microvascular complications of T2DM and the most common cause of end-stage renal disease [1,2]. Despite the continuous increase in the prevalence of DN globally, its pathogenesis has not been fully elucidated, and highly effective treatments remain inadequate. MicroRNAs (miRNAs) are small noncoding RNA molecules that regulate gene expression and are involved in the onset and development of tubular interstitial sclerosis and end-stage glomerular lesions that occur in various forms in CKD [3]. Therefore, miRNAs are potential therapeutic targets for DN.

The miR-92 family is one of the important miRNA families and involved in the regulation of tumor proliferation, apoptosis, invasion, and metastasis [4–7]. Yang et al. found that miR-92d-3p can regulate the complement pathway by targeting complement component 3 (C3) and thereby control acute immune response to bacterial infection [8]. Qiang et al. found that inhibiting miR-92d-3p expression in Nile tilapia head kidney can cause a significant increase in C3 expression and subsequent increase in the mRNA expression levels of interleukin (IL) 1β (IL-1β), tumor necrosis factor-α (TNF-α), and interferon-γ (INF-γ) and white blood cell count; thus, inhibited miR-92d-3p expression promotes an inflammatory response in Nile tilapia infected with *Staphylococcus aureus* [9]. miR-92d-3p may regulate immune inflammatory response by regulating the activity of C3, but the exact mechanism is unknown.

The deposition of C3, as a member of the complement system in glomeruli, is considered an important cause of various kidney diseases [10]. The activation of the glomerulus complement system in diabetic patients can exacerbate glomerular injury, and an injured glomerulus further activates the complement pathway [11]. C3 is an acute phase reactant and has an important role in the activation of the complement system. Additionally, C3 may play a role in the development of microvascular diseases [12]. In kidney diseases, the deposition of C3 occurs in the glomeruli and glomerular capillaries of animal models. Proteinuria and glomerular damage in mice can be alleviated by inhibiting C3 deposition [13].

HMGB1 can bind to cell surface receptors (such as the receptor of advanced glycosylation end-product [RAGE] and toll-like receptor 4) and may play an important role in promoting TGF-β1 production in DN and renal fibrosis [14]. The inhibition of the C3/HMGB1/TGF-β1 signaling pathway can inhibit the progression of renal fibrosis in DN [15]. HMGB1 is a DNA-binding protein released by the nucleus into the extracellular fluid through active secretion or passive release, and as a damage-related molecular model molecule (DAMP) [16], can bind to cell surface receptors, such as toll-like receptors (TLR) and RAGE and play a role in promoting the expression of inflammatory cytokines, such as TNF-α, IL-1β, and TGF-β1 [17]. TGF-β1 is considered a key regulator of renal fibrosis [18]. Substantial evidence supports the role of TGF-β in the onset and development of renal fibrosis [19]. Meanwhile, the underlying pathology of tubulointerstitial fibrosis is epithelial-to-interstitial transition (EMT) or the transdifferentiation of renal tubular epithelial cells into fibroblasts.

Therefore, the present study aims to determine whether miR-92d-3p can target complement C3 to modulate the activity of the C3/HMGB1/TGF-β1 pathway, inhibit immune inflammatory response and EMT associated with DN, and prevent or slow down the progress of DN.

## Materials and methods

### Patients

A total of 42 patients treated in the Second Affiliated Hospital of Nanchang University from January to December 2018 were selected. Then, 20 of these patients, which had renal trauma and were undergoing nephrectomy, were included in the normal control group, and 22 patients, which had DN and underwent renal biopsy histopathology, were included in the DN group. All steps were carried out in accordance with the approved topic, and each participant signed written informed consent. DN was diagnosed according to the criteria described in the American Diabetes Association Guidelines published in 2014. The exclusion criteria were as follows: pregnancy/breastfeeding, type 1 diabetes or other types of diabetes, other types of secondary kidney diseases or those caused by primary kidney diseases, severe hypertension, or renal insufficiency caused by high blood pressure, and suffering from urinary tract infection. The present study was approved by the medical ethics committee of the College of Medicine of Jiangxi University of Technology and in line with the Declaration of Helsinki.

### Animals and exposure

All animal experiments were performed in the Animal Center College of Medicine of Jiangxi University of Technology. The animal experiment of the present study was approved by the animal ethics committee of the College of Medicine of Jiangxi University of Technology. Six-week-old male C57BL/6J mice weighing 24 ± 2 g were purchased from Beijing Huafukang Biotechnology Co., Ltd. and reared in an SPF animal house at 23°C in a 12-h day/12-h night cycle. Some of the mice were selected as normal controls and fed with a standard diet (8% fat). The other mice received a high-fat diet (HFD) (containing 40% fat) for 8 weeks. A single dose of STZ (30 mg/kg) of 50 mM citrate buffer (pH 4.0) was injected intraperitoneally into the HFD animals daily for 7 days, whereas the normal control group received an equal volume of citrate buffer. One week after the last injection, blood glucose levels were checked. Random blood glucose of ≥16.7 mmol/l was considered diabetes, and 24 h UMA of ≥30 mg was considered DN. Then, the DN mice were randomly divided into the DN model group (*n*=10), DN model + lentivirus NC (negative control) group (*n*=10), and DN model + lentivirus miR-92d-3p group (*n*=10). Throughout the experiment, all mice had free access to food and water. The mice were monitored daily, and blood was drawn from the tail vein through standard laboratory methods and used in determining plasma glucose. In addition, the mice received intraperitoneal injection of 0.5% pentobarbital sodium and were killed through carbon dioxide release device.

### Lentiviral transfection

DN mice were transfected with lentivirus overexpressing the miR-92d target gene (synthesized by Shanghai Genechem Technology Co., Ltd.). After abdominal disinfection, 20 mice were anesthetized with 40 mg/kg pentobarbital sodium. The abdominal cavity was opened from the midline of the abdomen until the kidneys were exposed. With a 29-gauge syringe, 150–200 μl of lentiviral vector solution (titer 1 × 10^6^ cfu/ml) was injected into the kidneys of the mice in the DN model + lentivirus miR-92d-3p group. The kidneys of the mice in the DN model + lentivirus NC group were injected with the same dose of saline. After the incisions were closed, the mice were returned to the cages. On the 28th day, the mice were killed, and the kidneys were removed. The kidney tissues were stored at −80°C.

### Cell culture and exposure

A renal tubular epithelial cell line (HK-2, American Type Culture Collection, Rockville, Maryland, U.S.A.) was cultured in a DMEM/F12 medium containing 10% FBS (Gibco), placed in an incubator containing 5% CO_2_ at 37°C, digested with 0.05% trypsin, and passaged at a ratio of 1:3 every 5 days.

The cell suspension was inoculated in a 12-well plate at a concentration of 3 × 10^5^ cells/ml and incubated in a constant temperature incubator at 37°C for 16–24 h. When the cell fusion rate reached 30%, the medium was changed, and HiTransG P infection enhancer and a medium containing lentiviruses (20 μl/well) were added. The resulting medium was replaced with a conventional medium after cultivation for 16 h, then cultivation was continued. After infection for 72 h, the medium was changed to a serum-free medium, and human recombinant TGF-β1 (10 ng/ml, Peprotech, U.S.A.) and human recombinant C3a (20 ng/ml, R&Dsystems, U.S.A.) were added according to different groups: ① solvent control, ② C3a, ③ C3a + mimics-NC, and ④ C3a + miR-92d-3p mimics. The expression levels of miR-92d-3p, C3, IL-1β, IL-6, TNF-α, HMGB1, TGF-β1, fibronectin, α-SMA, E-cadherin, and vimentin were determined.

### Western blotting

Total protein were extracted using a radio immunoprecipitation assay lysis buffer (Beyotime, Shanghai, China). The sample was processed through 12% polyacrylamide gel electrophoresis and then transferred on to the PVDF membranes, which were then blocked in 5% nonfat dried milk for 2 h at room temperature. After blocking, primary antibody fibronectin (Abcam, Cambridge, MA, U.S.A.), α-SMA (Abcam, Cambridge, MA, U.S.A.), vimentin (Abcam, Cambridge, MA, U.S.A.), E-cadherin (Abcam, Cambridge, MA, U.S.A.), and GAPDH (Abcam, Cambridge, MA, U.S.A.) were added to the membranes. The membranes were incubated at 4°C overnight and then with HRP-conjugated secondary antibody at 37°C for 2 h. Finally, a gel imager was used for analysis. GAPDH was used as an internal control. All experiments were performed in triplicate independently.

### Enzyme-linked immunosorbent assay

HK-2 cells were collected, and the levels of IL-1β, IL-6, and TNF-α in the cultured HK-2 cells of each group were detected using an enzyme-linked immunosorbent assay (ELISA) kit (TOPEL02180, Biotopped, Beijing, China). All the steps of the assay were performed according to the manufacturer’s instructions.

### qRT-PCR

Total RNA was extracted from the mouse kidney cortex or HK-2 cells, and a first-strand cDNA was constructed using a reverse transcription system kit (Thermo, U.S.A.). Exactly 1 μg of RNA was reverse-transcribed into cDNA with RT primers. The primers used were obtained from Genscript Corp (Nanjing, China). The sequences were as follows: miR-92d-3p: TATGGCACTTATCCCGGCC, C3: F: 5′-CAGGCAGGAGGATGTATCGG-3′; R: 5′-TGCCAGCGTCAAGTCTTTTCT-3, HMGB1: F: 5′-CCTCTCAGATTGCACCTCGT-3′; R: 5′-TATGTCGTCATCCCGCAGTC-3′, TGF-β1: F: 5′-GAGGCGGTGCTCGCTTTGTA-3′; R: 5′-CGTTGTTGCGGTCCACCATTA-3′, α-SMA: F: 5′-GCGTGGCTATTCCTTGGTTA-3′; R: 5′-TGATGCTGTTGTAGGTGGTTTC-3′, E-cadherin: F: 5′-CCCACCACGTACAAGGGTC-3′; R: 5′-CTGGGGTATTGGGGG CATC-3′, Col I: F: 5′-GTACATCAGCCCAAACCCCA-3′; R: 5′-CAGGATCGGAACCTTCGCTT-3′, GAPDH: F: 5′-GCTGAGTATGTCGTGGAGT-3′; R: 5′- GTTCACACCCATCACAAAC-3′, U6: RT primer: 5′-CGCTTCAGGAATTTGCGTGTCAT-3′; F:5′-GCTTCGGCAGCACATATACTAAAAT-3′; R: 5′-CGCTTCACGAATTTGCGTGTCAT-3′. The expression level of miRNA was standardized for U6, and the expression level of the target gene for target U6 was standardized. GAPDH was measured through the *C*_t_^(^^ΔΔ*C*_t_^^)^ method.

### Immunofluorescence

HK-2 cell slides were fixed, penetrated, and closed. Afterward, the slides were mixed with anti-fibronectin (1 μg/ml, Abcam, ab64693, U.K.), anti-α-SMA (1 μg/ml, Abcam, ab64693, U.K.), anti-E-cadherin, (1:200, Bioworld Technology, Inc., U.S.A.), and anti-vimentin (1:200, Bioworld Technology, Inc., U.S.A.) and incubated overnight. Then, the slides were incubated with secondary antibody (1:200, ZB-2306, ZSGB-Bio, Beijing, China) for 1 h according to the manufacturer’s instructions and analyzed with a fluorescence microscope.

### Statistical analysis

SPSS 19.0 was used for statistical analysis. All the values were expressed as mean ± standard deviation (SD). One-way analysis of variance (ANOVA) or LSD test was used in analyzing data. A *P*-value of <0.05 was considered statistically significant. The Pearson correlation coefficients of biological parameters related to miR-92d-3p levels were calculated.

## Results

### Expression of miR-92d-3p in the kidney tissues of patients with DN was down-regulated

**Figure 1:**
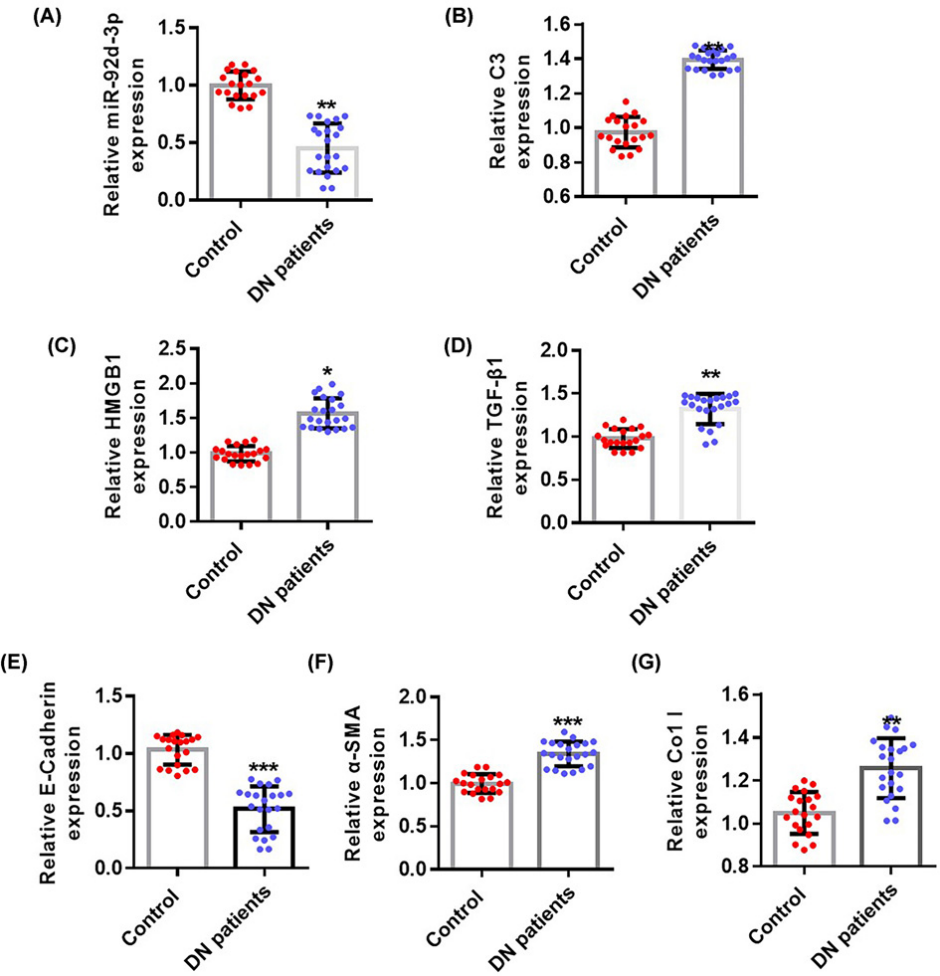
Expression levels of miR-92d-3p in the kidneys of patients with DN decreased qRT-PCR was used in detecting the expression levels of miR-92d (**A**), C3 (**B**), HMGB1 (**C**), TGF-β1 (**D**), E-cadherin (**E**), α-SMA (**F**), and Col I (**G**). miR-92d-3p was down-regulated in the renal tissues of patients with DN. The C3/HMGB1/TGF-β1 pathway was activated. Data were expressed as mean ± standard. Control group, *n*=20; DN group *n*=22. Compared with the control group, *P<0.05, **P<0.01, ***P<0.001. Student’s *t* test.

To investigate the role of miR-92d-3p in DN, we first explored the expression of miR-92d-3p in the kidney tissues of patients with DN through qRT-PCR. miR-92d-3p expression was significantly reduced compared with that in the normal control group (Figure 1A). Then, qRT-PCR was used in detecting the expression levels of C3 (Figure 1B), HMGB1 (Figure 1C), TGF-β1 (Figure 1D), E-cadherin (Figure 1E), α-SMA (Figure 1F), and Col I (Figure 1G). The expression levels of C3, HMGB1, TGF-β1, α-SMA, and Col I in the kidney tissues of patients with DN increased, whereas the expression levels of E-cadherin decreased compared with those in the normal control group.

### Expression of miR-92d-3p was down-regulated in the kidney tissues of DN mice

**Figure 2:**
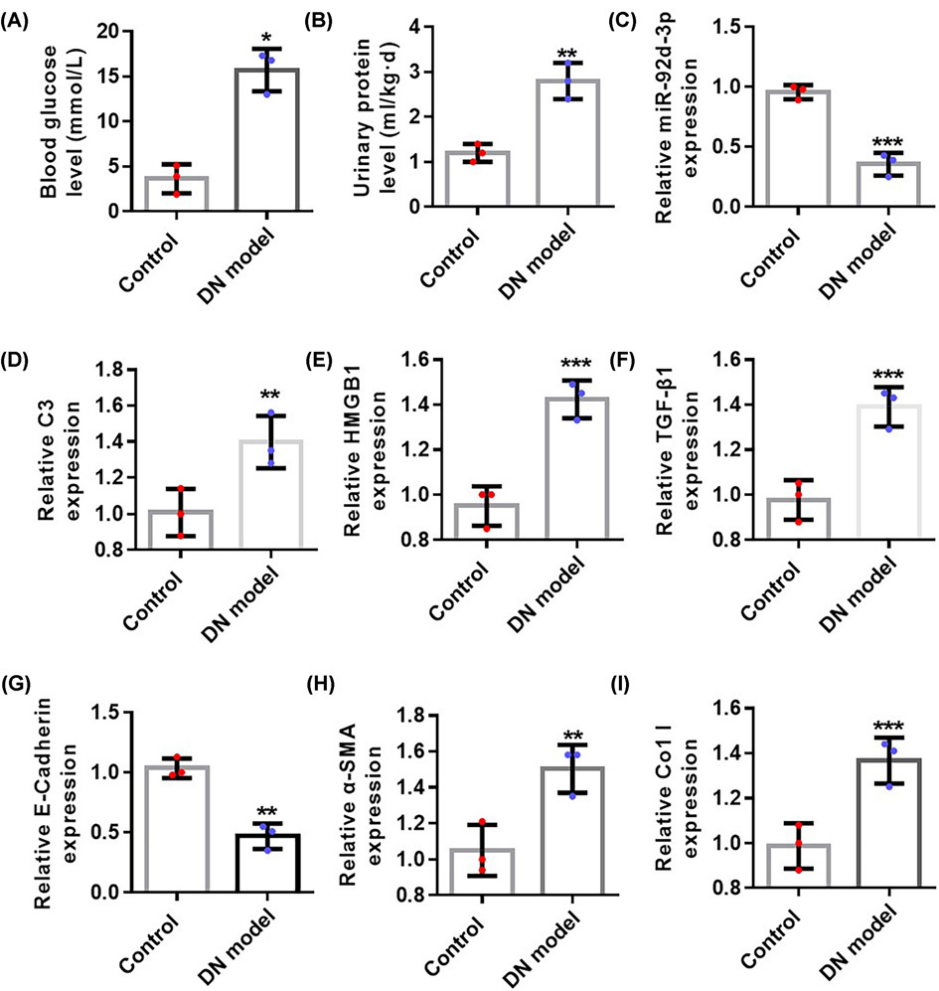
Expression levels of miR-92d-3p in the kidneys of DN mice decreased (**A**) Blood glucose level detection in the control and DN model groups. (**B**) Urine protein level detection in the control and DN model groups. (**C**) The expression of mir-92D was detected by qPCR. We used qRT-PCR to detect the expression levels of C3 (**D**), HMGB1 (**E**), TGF-β1 (**F**), E-cadherin (**G**), α-SMA (**H**), and Col I (**I**) in the kidney tissues of the two groups of mice. All values were expressed as mean ± standard, *n*=5. Compared with the normal control group, *P<0.05, **P<0.01, ***P<0.001. Student’s *t* test.

Blood glucose levels (Figure 2A) and urine protein levels (Figure 2B) significantly increased compared with those in the control group, indicating that DN mouse model was successfully established. qRT-PCR was then used in detecting the expression levels of miR-92d-3p (Figure 2C), C3 (Figure 2D), HMGB1 (Figure 2E), TGF-β1 (Figure 2F), E-cadherin (Figure 2G), α-SMA (Figure 2H), and Col I (Figure 2I) in the kidney tissues of two groups of mice. The expression levels of C3, HMGB1, TGF-β1, α-SMA, and Col I in the kidney tissues of DN mice increased, whereas the expression levels of miR-92d-3p and E-cadherin decreased compared with those in the normal control group.

### miR-92d-3p suppressed the progression of DN disease by inhibiting the activation of the C3/HMGB1/TGF-β1 pathway

**Figure 3:**
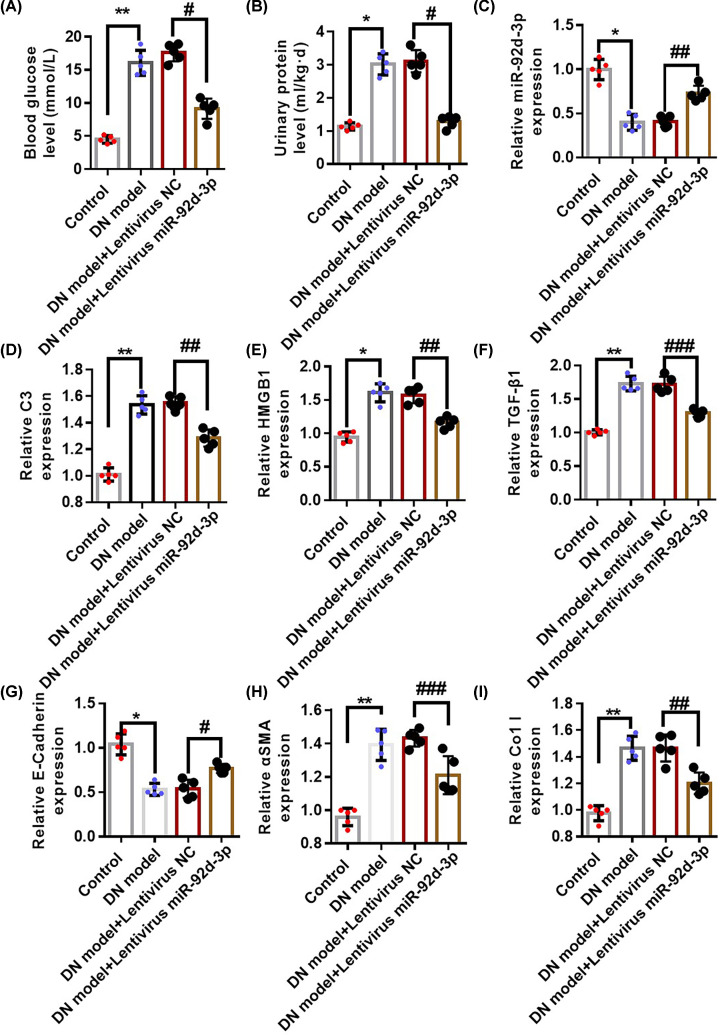
miR-92d-3p reduced the progression of DN renal fibrosis by inhibiting the activation of the C3/HMGB1/TGF-β1 pathway We overexpressed miR-92d-3p in the kidneys of DN mice through lentivirus transfection and detected changes in blood glucose (**A**) and urinary protein (**B**) in DN mice. We used qRT-PCR to detect the expression levels of miR-92d-3p (**C**), C3 (**D**), HMGB1 (**E**), TGF-β1 (**F**), E-cadherin (**G**), α-SMA (**H**), and Col I (**I**) in the kidney tissues of the mice in each group. Data were average ± standard (*n*=5 per group). *P<0.05, **P<0.01, ^#^*P*<0.05, ^##^*P*<0.01, ^###^*P*<0.001. One-way ANOVA.

To study the mechanism of miR-92d-3p, we overexpressed miR-92d-3p in the kidneys of DN mice through lentivirus transfection and detected changes in the blood glucose (Figure 3A) and urine protein levels (Figure 3B) of the mice. qRT-PCR was used in detecting the expression levels of miR-92d-3p (Figure 3C), C3 (Figure 3D), HMGB1 (Figure 3E), TGF-β1 (Figure 3F), E-cadherin (Figure 3G), α-SMA (Figure 3H), and Col I (Figure 3I) in the kidney tissues of the mice in each group. The degree of increase in the expression levels of C3, HMGB1, TGF-β1, α-SMA, and Col I in the kidney tissues of DN model + lentivirus miR-92d-3p group decreased, and the degree of decrease in the expression levels of miR-92d-3p and E-cadherin decreased compared with those in the DN model group.

### miR-92d-3p inhibited the production of C3-induced inflammatory factors in HK-2 cells

**Figure 4:**
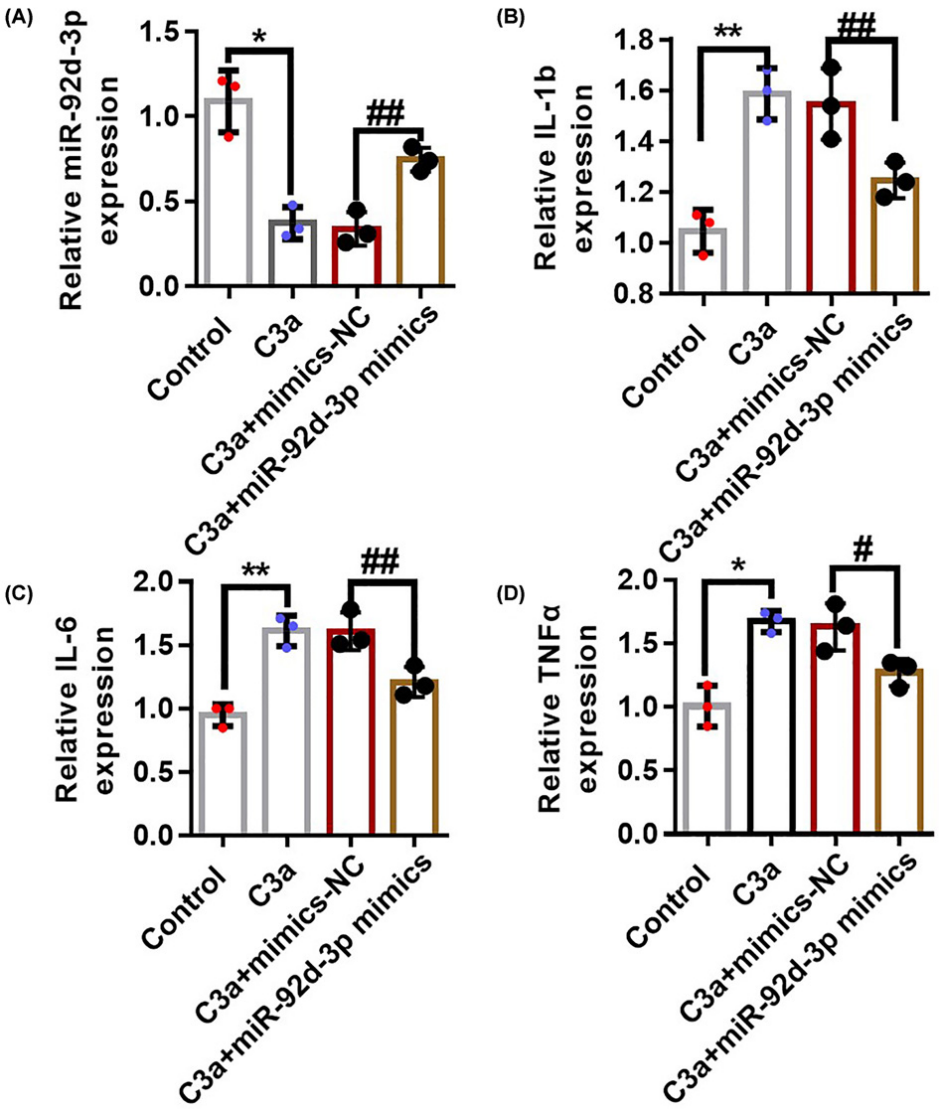
miR-92d-3p inhibited the production of C3-induced inflammatory factors in the HK-2 cells We used qRT-PCR to detect the expression level of miR-92d-3p in each group (**A**). We detected the production of IL-1β (**B**), IL-6 (**C**), and TNF-α (**D**) in each group through ELISA. Data were average ± standard. Three biological replicates per group. Compared with the normal control group, *P<0.05, **P<0.01; compared with the C3a+mimics-NC (NC: negative control) group, ^#^*P*<0.05, ^##^*P*<0.01. One-way ANOVA.

We transfected miR-92d-3p with lentivirus in cultured renal tubular epithelial HK-2 cells, then recombinant human purified C3 protein was added before the relationship between miR-92d-3p and C3 was observed. qRT-PCR was used in detecting intracellular miR-92d-3p expression in the NC, C3a, C3a + mimics-NC, and C3a + miR-92d-3p mimics groups. The expression levels of miR-92d-3p in the cells of the C3a and C3a + mimics-NC (negative control) groups decreased compared with those in the NC group, whereas the expression levels of miR-92d-3p in the C3a + miR-92d-3p mimics groups were higher than those in the C3a and C3a + mimics-NC groups but decreased compared with the miR-92d-3p expression level in the NC group (Figure 4A). We detected the production of IL-1β (Figure 4B), IL-6 (Figure 4C), and TNF-α (Figure 4D) in the cells in each group with ELISA kits. The results showed that the expression of inflammatory factors in the C3a and C3a + mimics-NC groups increased compared with that in the NC group, indicating that C3a can stimulate the production of inflammatory factors in HK-2 cells. The levels of inflammatory factors in the cells of the C3a + miR-92d-3p mimics group decreased compared with that in the C3a + mimics-NC group, indicating that miR-92d-3p inhibited the production of inflammatory factors promoted by C3a in the HK-2 cells.

### miR-92d-3p inhibited the activation of C3/HMGB1/TGF-β1 pathway and EMT induced by C3a in HK-2 cells

**Figure 5:**
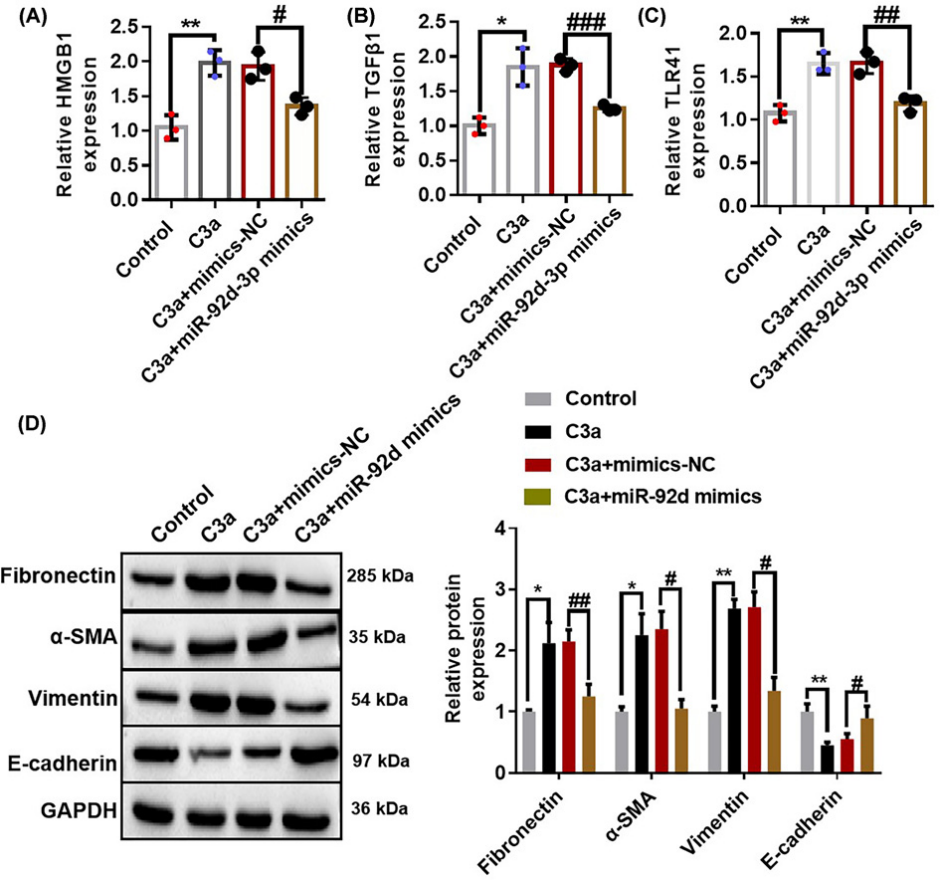
miR-92d-3p inhibited the activation of the C3/HMGB1/TGF-β1 pathway and EMT induced by C3a in the HK-2 cells We used qRT-PCR to detect the expression levels of HMGB1 (**A**), TGF-β1 (**B**), and TLR4 (**C**) in each group and performed Western blotting to detect the expression levels of fibronectin, α-SMA, vimentin, and E-cadherin (**D**). Data were expressed as mean ± standard. Three biological replicates per group. Compared with the normal control group, *P<0.05, **P<0.01; compared with the C3a+mimics-NC group group, ^#^*P*<0.05, ^##^*P*<0.01, ^###^*P*<0.001. One-way ANOVA.

qRT-PCR was used in detecting the expression levels of HMGB1 (Figure 5A), TGF-β1 (Figure 5B), and TLR4 (Figure 5C) in each group, and Western blotting were performed for the detection of the expression levels of fibronectin, α-SMA, vimentin, and E-cadherin (Figure 5D). The results showed that the expression levels of HMGB1, TGF-β1, TLR4, fibronectin, α-SMA, and vimentin in the C3a and C3a + mimics-NC groups increased, whereas the expression levels of HMGB1, TGF-β1, TLR4, fibronectin, α-SMA, and vimentin in the C3a + miR-92d-3p mimics group decreased compared with those in the NC group. The expression of E-cadherin showed an opposite result. All the above results suggested that overexpression of miR-92d-3p inhibited the activation of the C3/HMGB1/TGF-β1 pathway and EMT induced by C3a in the HK-2 cells.

### miR-92d-3p inhibited the activation of the C3/HMGB1/TGF-β1 pathway and EMT induced by TGF-β1 in HK-2 cells

**Figure 6:**
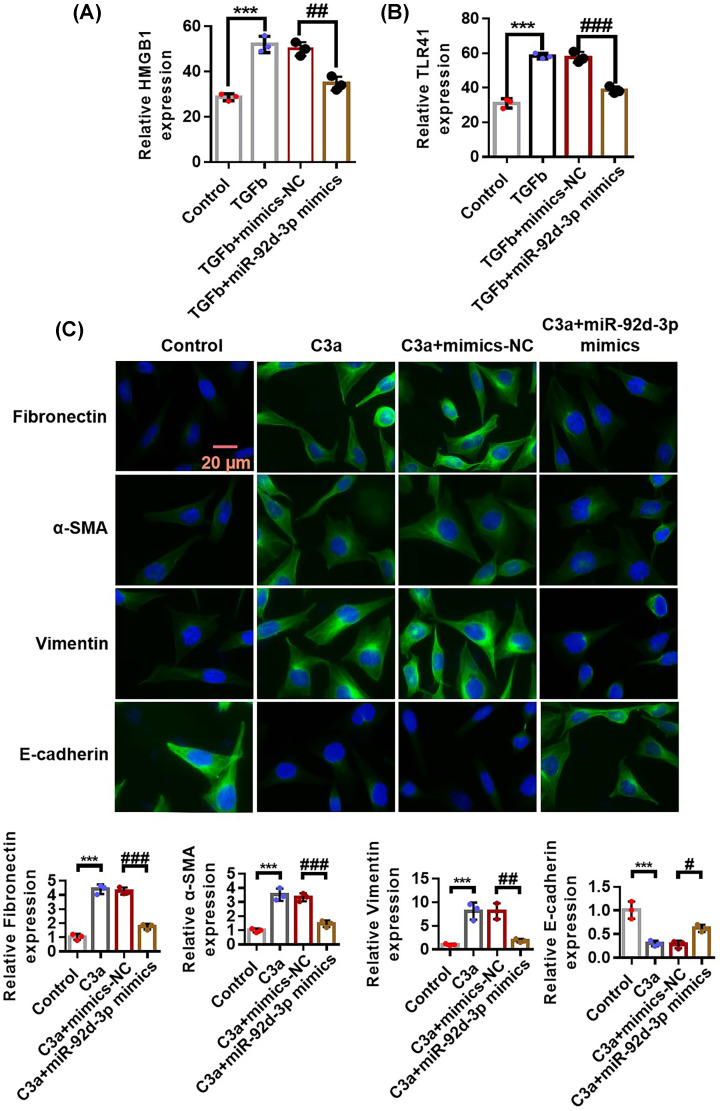
miR-92d-3p inhibits the activation of the C3/HMGB1/TGF-β1 pathway and EMT induced by TGF-β1 in the HK-2 cells (**A**) qRT-PCR to detect the expression levels of HMGB1 in each group. (**B**) qRT-PCR was used to detect the expression levels of TLR4 in each group. (**C**) Immunofluorescence experiments was performed for the detection of the expression levels of fibronectin, α-SMA, vimentin, and E-cadherin. Three biological replicates per group. Compared with the normal control group, ***P<0.001; compared with the C3a+mimics-NC group group, ^#^*P*<0.05, ^##^*P*<0.01, ^###^*P*<0.001. One-way ANOVA.

We transfected miR-92d-3p with lentiviruses in cultured renal tubular epithelial HK-2 cells and then added recombinant human purified TGF-β1 protein to observe the relationship between miR-92d-3p and TGF-β1. We used qRT-PCR to detect the expression levels of HMGB1 (Figure 6A) and TLR4 (Figure 6B) in each group and used immunofluorescence assays to detect the expression levels of fibronectin, α-SMA, vimentin, and E-cadherin (Figure 6C). The results showed that the expression levels of HMGB1, TLR4, fibronectin, α-SMA, and vimentin in the C3a and C3a + mimics-NC groups increased, and the expression levels of HMGB1, TLR4, fibronectin, α-SMA, and vimentin in the C3a+miR-92d-3p mimics group increased compared with those in the NC group. The expression of E-cadherin showed an opposite result. All the above results suggested that the overexpression of miR-92d-3p inhibited the activation of the C3/HMGB1/TGF-β1 pathway and EMT induced by TGF-β1 in the HK-2 cells.

## Discussion

T2DM is a serious health problem worldwide [20]. DN is one of the most important causes of end-stage kidney disease [21], affecting 15–25% of patients with T1DM and 30–40% of patients with T2DM. A cohort study conducted on 95202 people showed that the use of the Mendelian random allocation method in confirming high concentrations of C3 is associated with increased risk of DN. The determination of plasma C3 may play a central role in risk stratification and screening plan for prediabetic individuals [22]. C3 is highly expressed in the kidneys of DN rats and mainly expressed in the renal tubules. It is related to the progression of DN, and the activation of the complement system is a progressive factor of DN [23]. Inflammation is a common pathogenic mechanism of CKD [24,25]. In human and animal models, the increased expression of proinflammatory factors is related to the progression of DN [26]. The role of C3a in inflammatory response has been fully demonstrated, and the C3/HMGB1/TGF-β1 pathway activation plays a role in inflammatory response in tilapia head kidney infected by *Staphylococcus aureus* [9]. This process is closely related to the interaction between miR-92d-3p and C3. miR-92d-3p may play an important role in the onset of CKD and the occurrence and development of end-stage glomerular lesions. miR-92 plays an important role in the disease process of organ fibrosis. Wang et al. reported that cardiac-derived exosomes miR-92a mediate the activation of myofibroblasts after ischemia in vitro and in vitro [27,28]. Targeting miR-92d-3p is a potential therapeutic target for DN. Thus, studying the relationship of miR-92d-3p with the C3/HMGB1/TGF-β1 pathway and EMT in DN renal fibrosis is necessary.

Podocyte hypertrophy [29], apoptosis [30], and EMT [31] are the main causes of DN. HMGB1 induced by hyperglycemia may cause kidney damage in diabetic rats, and the pathogenic effect of HMGB1 may depend on the activation of RAGE and NF-κB [32]. HMGB1 is a damage-related molecular pattern that can be actively or passively released from various cells under different conditions and plays a key role in the pathogenesis of inflammation and angiogenesis-dependent diseases [33]. Additionally, HMGB1 is involved in the development of T2DM by inducing autophagy [34], and HMGB1 deletion can inhibit podocyte EMT by inhibiting TGF-β/smad1 signaling [35]. Thus, HMGB1, as a proinflammatory cytokine, may play an important role in the progression of DN by inducing increase in downstream TGF-β expression.

In the present study, the kidney tissues of patients with DN were first analyzed. The expression levels of miR-92d-3p decreased compared with miR-92d-3p expression in the control. Then, after the establishment of the DN mouse model, on the DN mouse model with overexpressed miR-92d in the kidneys and models of cell lines with overexpressed miR-92d-3p, mutual verification was conducted *in vitro* and *in vivo*. We found that the expression levels of miR-92d-3p in the kidneys of DN mice decreased, and the C3/HMGB1/TGF-β1 pathway was activated. However, the C3/HMGB1/TGF-β1 pathway was inhibited in the kidneys of DN mice with overexpressed miR-92d-3p. Moreover, the expression levels of markers for renal fibrosis, such as vimentin, α-SMA, and Col I decreased. The results of the *in vitro* cell experiments showed that the supplementation of recombinant purified protein C3a and TGF-β1 can promote the activation of the C3/HMGB1/TGF-β1 pathway and EMT. By contrast, the overexpression of miR-92d-3p can prevent the activation of the pathway and reduce EMT. These results indicated that miR-92d-3p can suppress the progression of DN renal fibrosis by inhibiting the activation of the C3/HMGB1/TGF-β1 pathway.

The present study has some shortcomings. The clinical application of miR-92d-3p, as a noncoding RNA, should be further explored. In the present study, only lentiviruses were used in overexpressing miR-92d-3p. The knocking down of miR-92D-3P is necessary in verifying the role of miR-92d-3p in fibrosis.

## Conclusion

During the progression of DN disease, miR-92d-3p interacts with C3 to inhibit the activation of the C3/HMGB1/TGF-β1 pathway and EMT, thereby preventing the progression of DN renal fibrosis. Our study may provide a theoretical basis for understanding the pathogenesis and treatment of DN.

## Data Availability

All the data presented in the present study are available from the corresponding author upon reasonable request.

## Abbreviations

CKD: chronic kidney disease
DN: diabetic nephropathy
ELISA: enzyme-linked immunosorbent assay
EMT: epithelial-to-interstitial transition
HFD: high-fat diet
IL: interleukin
miRNA: microRNA
RAGE: receptor of advanced glycosylation end-product
TLR: Toll-like receptor
TNF-α: tumor necrosis factor-α
T2DM: type 2 diabetes
